# Operationalizing Pandemic Vaccinations at a Regional Supermarket Chain Pharmacy

**DOI:** 10.1017/dmp.2021.43

**Published:** 2021-02-16

**Authors:** Chiara A. Gessler, Reneé M. Richardson, Deanne L. Hall, Kim C. Coley

**Affiliations:** 1 Giant Eagle Pharmacy and Duquesne University, Pittsburgh, PA; 2 Community Partnerships, Giant Eagle Pharmacy, Pittsburgh, PA; 3 Pharmacy and Therapeutics, University of Pittsburgh School of Pharmacy, Pittsburgh, PA

**Keywords:** community pharmacy, pandemics, vaccination, emergency preparedness, pharmacists

## Abstract

Community pharmacies were underutilized as vaccination locations during the 2009 H1N1 pandemic. Since that time, community pharmacies are a common location for seasonal influenza vaccinations with approximately one-third of adults now getting vaccinated at a pharmacy. Leveraging community pharmacies to vaccinate during a pandemic such as pandemic influenza or the current coronavirus disease (COVID-19) pandemic will result in a more timely and comprehensive public health response. The purpose of this article is to summarize the results of a strategic planning meeting held in 2017 that focused on operationalizing pandemic influenza vaccinations at a regional supermarket chain pharmacy. Participating in the planning session from the supermarket chain were organizational experts in pharmacy clinical programs, managed care, operations leadership, supply chain, information technology, loss prevention, marketing, and compliance. Additionally, experts from the county and state departments of health and university faculty collaborated in the planning session. Topics addressed included (1) establishing a memorandum of understanding with the state, (2) developing an internal emergency response plan, (3) scaling the pandemic response, (4) considerations for pharmacy locations, (5) staffing for pandemic response, (6) pandemic vaccine-specific training, (7) pharmacy workflow, (8) billing considerations, (9) documentation, (10) supplies and equipment, (11) vaccine supply chain, (12) communications, and (13) security and crowd control. Information from this planning session may be valuable to community pharmacies across the nation that seek to participate in COVID-19 pandemic vaccinations.

## Introduction

During the 2009 H1N1 pandemic, community pharmacies were underutilized as vaccination locations, with only 10% of adults receiving an H1N1 vaccine at a pharmacy.^1^ However, over the last decade, community pharmacies have become a common location for seasonal influenza vaccinations. In 2018, approximately 32% of adults reported getting their seasonal influenza vaccination at a pharmacy.^2^ There are more than 60,000 community pharmacies in the United States and many Americans live near a community pharmacy.^3,4^ Engaging additional community partners, such as pharmacists, may result in a more timely and comprehensive public health response during pandemics.^5^ Schwerzmann and colleagues^6^ modeled the effect of community pharmacy vaccinators during a future influenza pandemic. They found that by using community pharmacies, vaccination coverage nationally was reduced by 7 weeks.^6^

In order to expand the capacity for pandemic influenza vaccination through community pharmacies in western Pennsylvania, key public health officials from Allegheny County and the Pennsylvania Department of Health (PA DOH) and pharmacy leadership from Giant Eagle, Inc., the region’s largest supermarket chain pharmacy, were invited to participate in a 2-day strategic planning meeting in 2017. One goal of this meeting was to facilitate the development of a PA DOH Pharmacy Pandemic Memorandum of Agreement that could be used across the commonwealth.^7,8^ These agreements are not legally binding but are an important first step in engaging community pharmacies in the provision of pandemic influenza vaccines. They do not, however, provide guidance on how to operationalize pandemic vaccinations within the pharmacy.^9,10^ Therefore, a second goal, and the focus of this manuscript, was to develop a plan to operationalize pandemic influenza vaccinations at this regional supermarket chain pharmacy in Pennsylvania. Specifically, the team set out to explore scaling the pandemic response, pharmacy staffing and workflow, pandemic vaccine-specific training, managing vaccine supply, inventory and cold chain, documentation and billing, communications, and security.

Participating in this planning session from the supermarket chain were experts in pharmacy clinical programs, managed care, operations management, supply chain, information technology, loss prevention, and compliance. Additionally, experts in public health, pandemic planning, and immunization registries from the county and state departments of health were present. Finally, university faculty with expertise in public health, community pharmacy practice, and immunizations collaborated in the planning session. The session was mediated by a senior university faculty member with experience in meeting facilitation. Notes were taken by 2 meeting participants and consolidated into a final meeting report after feedback and input from meeting participants. Session topics were selected based on past experience with pandemic immunizations, as well as implementation and execution of mass immunization events. Information from this planning session may be valuable to all types of community pharmacies, such as traditional chain and independent pharmacies that seek to participate in large scale vaccination programs during pandemics, including the current coronavirus disease (COVID-19) pandemic.

## Establishing a Memorandum of Understanding (MOU) With the State

In 2018, the Association of State and Territorial Health Officials published a *Memorandum of Understanding (MOU) Toolkit for Public Health Agencies and Pharmacies* that was developed with the input from state health agencies and pharmacy stakeholders.^11^ As a result, many state departments of health have used this toolkit to develop their own MOU for influenza pandemics and other vaccine-related public health emergencies.^8^ Completing an MOU is an important first step for community pharmacies to begin the process of pandemic vaccination preparation. The MOU outlines the responsibilities for both the pharmacy and the state and opens communication channels essential to operationalize pandemic vaccinations.

## Internal Emergency Response Plan

Pharmacy organizations should implement an internal emergency response plan (ERP) in advance to facilitate preparation of a future pandemic.^12,13^ The ERP guides the pharmacy’s response outlined in the MOU and identifies key stakeholders that would be involved in activating the pandemic response. Elements of the ERP include (1) an executive summary that outlines the purpose and intent of the plan; (2) a definition of the pandemic that the ERP is intended to respond to; (3) identification of a Pandemic Management Team (PMT) and members’ roles and responsibilities related to the ERP; and (4) activation levels that define when the PMT should be alerted or when a pandemic response is necessary. The PMT should consist of a leader, a process guardian, and other key stakeholders. The leader will oversee the PMT and should be an individual with a leadership role in the organization. This individual will develop and understand all of the plans of the PMT, define the authority granted to the PMT, and provide guidance to the PMT. The process guardian will oversee the establishment of the MOU with the state and maintain open lines of communication with all state and local departments of health contacts. The process guardian will also be responsible for keeping the ERP up to date and notifying the leader of the PMT when a pandemic occurs. The process guardian is the facilitator of the ERP and members of the PMT. Other members of the PMT may include those of pharmacy operations, clinical pharmacy, information technology, supply chain, marketing, managed care, security, compliance, training, legal, and communications. If the pharmacy operates within a larger entity such as a grocery store, the response team should include members of other business units that may be affected by a pandemic response. Independent pharmacies that may not operate with a large support staff could consider the key components outlined below in preparation for a pandemic and development of their ERP. The ERP should address each of the items outlined below. All key stakeholders should be familiar with their responsibilities and the overall response outlined in the ERP allowing for immediate engagement and activation in the case of a pandemic.

## Scaling the Pharmacy’s Pandemic Response

The scale of the response depends on several variables that will not be known prior to the pandemic, such as the epidemiology of the pandemic and availability of the vaccine. Epidemiological data provided by the department of health will help identify geographic areas at highest risk for pandemic infections. This will aid in determining whether the response should involve a small number of pharmacies, possibly clustered in targeted geographic regions, or a large number of pharmacies covering a greater geographic area. Epidemiological data and vaccine availability will also inform the target population for vaccination. Pharmacies can use the demographic information that they have on their patient population to determine whether they service a large number of at-risk patients. Pharmacies with a high number of patients in the target population may be prioritized to receive pandemic vaccines. Another important factor in determining a pharmacy’s pandemic response is the amount of a vaccine available for distribution. If there is a large amount of a vaccine available, a greater number of pharmacies will be able to receive a vaccine in large amounts. However, if there is only a limited supply of a pandemic vaccine, pharmacies may receive a smaller allocation, or fewer pharmacies overall may receive the vaccine. Finally, factors such as vaccine storage capabilities, staffing needs, and pharmacy workflow configurations affect each location’s capacity for pandemic vaccine administration.

## Considerations for Pharmacy Locations

For pharmacy chains, a component of the internal response plan is the assessment of pharmacy locations and their suitability for a pandemic response based on geographical location and store layout. Hub pharmacies centrally located within regions should be identified, and these stores can then be further assessed for their ability to accommodate large patient volumes. Pharmacies that are located at the intersection of major roadways or on mass transit routes may be preferred vaccination locations because they are easily accessible to both the general public as well as staff members in the surrounding area. Those with large parking lots may offer space for traffic flow or outdoor areas for drive-through vaccinations. In addition to the physical location and outdoor space, store design also factors into the selection criteria. Stores with adequate space for all vaccine clinic functions, such as those with conference rooms or large open areas, should be considered before those with less usable space. High volume vaccine clinics require dedicated space for multiple stations and waiting areas, as well as separate entry and exit doors to facilitate patient flow.^14^ Additionally, during highly infectious pandemics, such as COVID-19, more space may be needed to accommodate social distancing of patients waiting to get vaccinated. Pharmacies may want to consider using outdoor areas or drive-through clinics to provide vaccinations under the circumstances of a highly contagious pandemic. Alternatively, holding an off-site clinic at a local facility may provide the space needed for clinic workflow and social distancing. Perhaps community centers or rented, public spaces may be available for an off-site clinic if adequate space is not available at the pharmacy location. Finally, pandemic vaccine storage capacity at the location is an important consideration. Pharmacies with existing adequate storage space for a vaccine, or with refrigeration or freezer space that can be reconfigured to support a clinic, may be preferred locations.

## Staffing for a Pandemic Response

Pharmacies serving as pandemic vaccination sites will need to be staffed adequately to provide vaccinations as well as maintain normal pharmacy operations. Pharmacies should plan for up to a 30–40% reduction in staff availability due to pandemic-related illness among team members or their family.^15^ Larger pharmacy chains have the resources to use floating pharmacists, casual pharmacists, and pharmacists from nearby locations to perform pharmacist-only duties. Floating pharmacy technicians, student pharmacist interns, front-end team members, and support staff may be used to perform non-pharmacists duties to maintain pharmacy operations or pandemic vaccine clinic operations. Local pharmacy schools could be engaged to provide student pharmacists to support the pharmacy’s pandemic operations. Access to student pharmacists may be even more important for small independent pharmacies with small staffs. Pharmacies may need an affiliation agreement with each pharmacy school to enlist student pharmacist volunteers in support of pandemic vaccine efforts at their sites.

## Training

Individuals involved in the pandemic vaccination process should be adequately trained on their role and the process. Based on the Centers for Disease Control and Prevention (CDC) guidelines, staff who are trained and involved in the response should get the vaccine and prophylactic treatment if available.^16^ Pharmacists and pharmacy staff involved in the provision of pandemic vaccines should receive infection control training as well as training on the appropriate use of personal protective equipment (PPE). Since there may be a limited initial supply of a pandemic vaccine, training in proper storage and handling is important so the vaccine is not wasted. Training on the state immunization registry may also be required and can be completed prior to a pandemic. These registries will be used for ordering pandemic vaccine and inventory management. Many states, including Pennsylvania, offer online training for their respective immunization registries. Finally, pharmacy staff should be trained on the pandemic vaccine product itself, including the recommended dose, route, schedule, target population, precautions, contraindications, and storage. In the United States, much of this training will be supplied by the CDC. For example, the CDC currently offers online training on the proper storage and handling of vaccines.

## Pharmacy Workflow

Depending on the volume of patients expected, pharmacies may administer pandemic vaccines within their current prescription workflow or provide vaccines outside of the workflow in a clinic setting. The CDC provides helpful guidance for planning vaccinations at temporary, satellite, or off-site clinics.^14^ The process chosen should have the least impact on business continuity and allow for the routine dispensing of medications, as well as the provision of other patient care services. These routine services may need to be adapted, however, to protect pharmacy staff and patients during a pandemic. In the case of providing routine vaccinations during the COVID-19 pandemic, the CDC has developed guidance that pharmacies can follow to support the safe administration of routine vaccines.^17^ In order to maximize patient access to pandemic vaccinations, as well as other pharmacy services, and limit interruptions in the current pharmacy workflow, pharmacies may need to implement a scheduling system or extend operation hours. If large volumes of patients are expected, pandemic vaccination clinics may be a better option to safely and effectively administer vaccines to patients. Vaccination clinics may occur at the pharmacy or at an alternative site near the pharmacy (eg, parking lot) or at an off-site location (eg, fire hall, local business, nursing home). Off-site vaccination clinics may be necessary for small pharmacies that do not have the ability to social distance large numbers of patients within their building. Use of a scheduling system will also facilitate management of large crowds. To efficiently move patients through the vaccine clinic, pharmacies can establish stations for specific tasks, such as vaccine eligibility screening, patient registration, medical screening, billing, vaccination administration, and post-vaccine observation. If adjudication of vaccine claims within clinic workflow is not feasible, establish a process to enter claims at a later time that will adhere to documentation and reporting requirements. In the case of a multi-dose vaccine, incorporate a process into the pharmacy and clinic workflow to include scheduling and notification of the follow-up dose(s).

## Vaccine Billing Considerations

All pandemic vaccines administered should be documented in the patient’s pharmacy record and reported according to state and federal requirements for pharmacy-administered vaccinations. Pharmacies may require prescription adjudication for this process to occur. Since pandemic vaccines are provided by the state at no cost, pharmacies are prohibited from charging the patient or a third party for the vaccine product. Pharmacies may be able to charge an administration fee to cover operational costs of providing pandemic vaccine services. The administration fee could potentially be paid by the patient’s insurer and may not exceed the regional Medicare vaccine administration rate. Pharmacies may not withhold the provision of pandemic vaccines supplied by the United States Government due to a patient’s inability to pay. Pharmacies may consider establishing contracts with third-party payors to bill for pandemic vaccine administration. Administration fee structures would not be included in the established MOU with the state.

## Documentation

Documentation and communication are key tenets of collaboration within the health care team to provide patient care. Pharmacists should provide documentation of pandemic vaccine administration to the patient, their medical provider, and to the state immunization registry. Patient documentation can be in the form of a copy of the vaccine screening and administration form and/or a vaccine record card. A vaccine record card will also be helpful to patients under circumstances of a multiple dose vaccine since it can provide them with information on when to return for subsequent doses. Additionally, it is recommended that the pharmacy have a process in place to contact patients to receive subsequent doses of vaccine if appropriate. In addition to documenting pandemic vaccine administration in the pharmacy’s records, pharmacies must also record this information in the appropriate state immunization registry. This will not only enable the information to be accessible to all health care providers, but also provide valuable data for public health entities tracking vaccination. In the event of a multiple dose pandemic vaccine series, any health care provider will be able to query the state immunization registry for an individual patient record. That information can be used to support real-time clinical decisions regarding the patient’s pandemic vaccine immunization status. While there are certain state-specific requirements to access the immunization registry, there may be several avenues to accomplish this. Pharmacies may manually document each patient record on the registry website or they may elect to use an electronic feed for automatic submission of patient records. In addition to housing immunization records, the state registry also serves the important function of inventory management. The state registry may be used during a pandemic to request vaccine supply and to report current levels of inventory. Each pharmacy may request vaccine supply weekly from the state’s allocation. Doses subsequently administered at each location are recorded and monitored via the state registry at least weekly, and each dose received must be accounted for and tracked. In the event of an adverse reaction to the pandemic vaccine, pharmacies should have a procedure in place to submit a report to the Vaccine Adverse Event Reporting System and to notify the patient’s medical provider.^18^

## Supplies and Equipment

Most of the supplies and equipment pharmacies need to provide pandemic vaccines mirror what is used for routine pharmacy vaccinations; however, some supplies may come from alternative sources. During a pandemic, it is expected that administration supplies such as alcohol swabs, syringes, and other supplies would be provided by the United States Government, in addition to the vaccine product. PPE such as masks and gowns may also be required for store and pharmacy personnel for infection control and may be provided with the administration supplies. It is recommended that pharmacies determine which supplies will be provided by the government and have a checklist of additional supplies needed. Pharmacies should also identify sources for these items in advance. It is expected that screening forms, vaccine information statements or other required patient education for pandemic vaccine products will be developed and made available by the proper government authorities.

Since there is a high likelihood that a pandemic vaccine would require refrigerated or frozen storage conditions, pharmacies should also assess their capacity to store large amounts of vaccine on-site. This is particularly important if the pandemic is occurring during influenza season since additional storage may be necessary to manage both the pandemic and influenza vaccines. In supermarket pharmacies, alternative refrigeration or freezer units within the grocery location may be used to properly store large amounts of a pandemic vaccine. If the pharmacy plans to provide vaccine at an off-site clinic, the cold chain can be maintained with qualified portable containers, packing materials, and a temperature-monitoring device. All fixed and portable refrigeration units and containers used for the storage and transport of a pandemic vaccine should meet the criteria set forth in the CDC’s Vaccine Storage and Handling Toolkit.^19^

Additional equipment may be necessary to support the vaccination process and flow at the pharmacy location. For example, an area with tables and chairs will be needed for patient screening for vaccine eligibility and completing other vaccine-related paperwork. An observational area with additional chairs can be used to monitor patients for adverse reactions post-vaccination. To control the flow of people, stanchions/bank ropes may be needed to section off areas of the store and support line formation. Large pharmacy locations may have space within the store that can accommodate these materials while maintaining social distancing requirements. Spaces outside of the pharmacy building, such as a parking lot, can also be considered in order to provide adequate space.

## Pandemic Vaccine Supply Chain

Pandemic vaccine is procured from the federal supply through the state Department of Health using the statewide immunization information system. This is in contrast to other vaccines that may be procured from manufacturers or distributors. Pandemic vaccines can be directed to the end-user pharmacy location or to a central location such as a warehouse for a pharmacy chain. If shipped to a central location, the pandemic vaccine can then be stored and distributed via established channels to pharmacy locations following cold chain procedures. Utilization of a central location provides some benefits for a large pharmacy chain, including expanding vaccine storage capacity and providing flexibility to meet individual pharmacy inventory needs. This avoids overwhelming the individual pharmacy location’s available storage space with a large, weekly supply from the state, and instead converts it to a smaller daily shipment from the central location. If a central facility is used for pandemic vaccines, a procedure should be implemented to separate pandemic supply from other vaccine supply, or another state’s vaccine supply if housed in the same facility. During a rapidly evolving pandemic, procedures should also be implemented for vaccine transport between pharmacy locations, return transport to the central location, or return transport to the state’s stockpile for redeployment to other locations. Cold chain procedures may involve an internal delivery truck or an outside courier using refrigerated cooler totes that employ a “cold chip” to report temperature excursion to the end user. All stakeholders should be involved in establishing the procedures for vaccine return transport given the cost and complicated logistics.

## Communication: External and Internal

External communication with the public should provide pandemic information consistent with that of the state health department as well as details about how patients may obtain a vaccine. This may include information on vaccine availability, clinic times, and clinic locations. This information can be shared in a timely manner via the pharmacy website, perhaps providing an interactive map to highlight stores within a pharmacy chain that have a vaccine. There may also be an opportunity for a searchable locator on the state Department of Health website. Websites may also include a pre-screening checklist or interactive tool to screen patients seeking vaccine and to provide appointment scheduling services for an appointment-based model. Pharmacies that use an app can program it so that patients receive notifications on pandemic vaccine availability. Social media posts and e-mail blasts can also be leveraged to advertise pandemic vaccine clinic schedules and locations. The pharmacy phone answering system could provide information to patients calling the pharmacy. Consider establishing a centralized hotline with existing customer service staff or offering the department of health hotline. In-store signage could inform current patients visiting those stores. Television, print, and radio advertisement, similar to community pharmacy marketing during flu season, could support the overall communication efforts with the public. Pharmacies may need to include messaging to patients to address patient concerns with vaccine safety and effectiveness. The CDC currently provides resources to address vaccine hesitancy, such as the “Vaccinate with Confidence” campaign.

Internal communications that engage both the pharmacy staff as well, as in the case of a supermarket pharmacy, staff from the entire store, are necessary for the success of a pandemic vaccine program. Communication algorithms and responsibilities should be outlined in the ERP. Communication strategies should include educating and training entire store team members around pandemic vaccine operations and how to engage with the community. Team members anywhere within the location should be equipped with talking points to respond to common customer inquiries. Store personnel may also be used to assist with crowd control and other non-pharmacist required tasks for pandemic vaccine clinics or to maintain normal operations of the pharmacy. Regular communication should occur to the entire store as new information becomes available surrounding the pandemic vaccination services offered.

## Security and Crowd Control

Pharmacies providing pandemic vaccinations should consider a multi-pronged approach for security and crowd management. For a large walk-in clinic setting, traffic control may be needed for the roads surrounding the pharmacy and the pharmacy’s parking area. Pharmacies should communicate with local law enforcement officials in advance for support with traffic flow. Traffic management may also be needed for clinics offering drive through vaccination services. Communicating with law enforcement is also vital for smaller pharmacies with limited parking or those located on busy streets.

In order to reduce crowding, staff should be available to communicate vaccine availability and wait times to patients prior to entering the vaccine clinic area. To minimize unnecessary travel, pharmacies can communicate which target populations are eligible to receive the vaccine. Use of an online pre-screening tool can help reduce the number of non-vaccine eligible patients at the vaccine clinic. Use of crowd control staff such as internal security personnel may be warranted to direct patients inside the vaccine clinic location. Patients may be formed into lines to complete the initial screening and given an estimated wait time for vaccine administration. Pharmacies should consider using a system that enables patients to leave and return at a scheduled time. Numbered tickets, such as those from a deli or bakery in the store, have been used in the past at supermarket pharmacies. A system to record waiting patients’ cell phone numbers to send text notifications for return may also be employed. Bottlenecks in the clinic workflow may be avoided through the use of additional staff to aid patients in completing the eligibility and medical screening forms. In addition, provisions should be made for patients with special needs to assist them through the clinic process in an efficient manner. In addition to crowd control, security should also be a consideration. Safeguards should be employed to ensure the safety of patients, staff, their belongings, and pandemic vaccine supply. Using bank ropes or a numbering system may prevent patients from cutting in line. Security personnel should be on hand to mediate any disputes. Finally, a pandemic vaccine should be in possession of the immunizing pharmacists and kept close at hand.

## Limitations

There are some considerations and potential limitations of the information described herein. First, as stated earlier, this planning exercise focused on supermarket chain pharmacies so much of the information generated related to pharmacies with greater resources such as space and staffing. Effort was made, however, to highlight opportunities for smaller pharmacies. Also, this work was based on a planning conference with experts from the Commonwealth of Pennsylvania and may not reflect issues that pharmacies located in other states or countries may experience.

## Conclusion

Using community pharmacies to support vaccination efforts during a pandemic will result in a more timely and comprehensive public health response. To be most effective, pharmacies need to consider all aspects of the pandemic vaccination response, ideally prior to a pandemic, through the establishment of an MOU with the state and a corresponding internal ERP. More specific planning details are dependent on the pandemic itself and are unknown until it arises. The information learned from this pandemic vaccine strategic planning session with a supermarket pharmacy provides pharmacies of all types with a general roadmap to pandemic vaccine preparedness.
